# Sequencing of transcriptomes from two *Miscanthus* species reveals functional specificity in rhizomes, and clarifies evolutionary relationships

**DOI:** 10.1186/1471-2229-14-134

**Published:** 2014-05-18

**Authors:** Changsoo Kim, Tae-Ho Lee, Hui Guo, Sung Jin Chung, Andrew H Paterson, Do-Soon Kim, Geung-Joo Lee

**Affiliations:** 1 Plant Genome Mapping Lab, University of Georgia, 111 Riverbend RD, Athens, GA 30602, USA; 2 Department of Horticulture, Chungnam National University, Daejeon 305-764, South Korea; 3 Department of Plant Science, Research Institute of Agriculture and Life Sciences, Seoul National University, Seoul 151-921, South Korea; 4 Present address: Genomics Division, National Academy of Agricultural Science, Rural Development Administration, Suwon 441-707, South Korea

**Keywords:** *Miscanthus sinensis*, *Miscanthus sacchariflorus*, Expressed sequence tags, Synonymous substitution, Orthologous sequences, Nonsynonymous SNPs

## Abstract

**Background:**

*Miscanthus* is a promising biomass crop for temperate regions. Despite the increasing interest in this plant, limited sequence information has constrained research into its biology, physiology, and breeding. The whole genome transcriptomes of *M. sinensis* and *M. sacchariflorus* presented in this study may provide good resources to understand functional compositions of two important *Miscanthus* genomes and their evolutionary relationships.

**Results:**

For *M. sinensis*, a total of 457,891 and 512,950 expressed sequence tags (ESTs) were produced from leaf and rhizome tissues, respectively, which were assembled into 12,166 contigs and 89,648 singletons for leaf, and 13,170 contigs and 112,138 singletons for rhizome. For *M. sacchariflorus*, a total of 288,806 and 267,952 ESTs from leaf and rhizome tissues, respectively, were assembled into 8,732 contigs and 66,881 singletons for leaf, and 8,104 contigs and 63,212 singletons for rhizome. Based on the distributions of synonymous nucleotide substitution (Ks), sorghum and *Miscanthus* diverged about 6.2 million years ago (MYA), *Saccharum* and *Miscanthus* diverged 4.6 MYA, and *M. sinensis* and *M. sacchariflorus* diverged 1.5 MYA. The pairwise alignment of predicted protein sequences from sorghum-*Miscanthus* and two *Miscanthus* species found a total of 43,770 and 35,818 nsSNPs, respectively. The impacts of striking mutations found by nsSNPs were much lower between sorghum and *Miscanthus* than those between the two *Miscanthus* species, perhaps as a consequence of the much higher level of gene duplication in *Miscanthus* and resulting ability to buffer essential functions against disturbance.

**Conclusions:**

The ESTs generated in the present study represent a significant addition to *Miscanthus* functional genomics resources, permitting us to discover some candidate genes associated with enhanced biomass production. Ks distributions based on orthologous ESTs may serve as a guideline for future research into the evolution of *Miscanthus* species as well as its close relatives sorghum and *Saccharum*.

## Background

Concerns about global warming in combination with increased use of fossil fuel have spurred growing interest in sources of renewable energy such as biofuels. The genus *Miscanthus* has been considered attractive as a feedstock for cellulosic biofuel production because the plants are adapted to temperate latitudes yet utilize the energy-efficient C4 photosynthetic pathway, producing high yields of biomass but with low-nutrient requirements, adaptation to marginal land due to resistance to abiotic stress, and not competing with use for food [1-3]. At present, 14 *Miscanthus* species are recognized, most of which are native to eastern Asia, but with a few found in Polynesia, the Himalayas, and southern Africa [4].

*Miscanthus* belongs to the Andropogoneae tribe of grasses, including many economically important crops such as maize, sorghum, and sugarcane. In particular, *Miscanthus* and sugarcane belong to the subtribe Saccharinae that may be characterized by its complex and high ploidy genomes. For example, *M. × giganteus* (2n = 3× = 57), which is specifically of interest due to its ability to accumulate biomass, is thought to result from crosses between *M. sacchariflorus* (2n = 4× = 76) and *M. sinensis* (2n = 2× = 38) [5]. Study of the large and complex *Miscanthus* genomes is challenging, although the whole genome sequence of closely-related sorghum (2n = 2× = 20) [6] provides a valuable reference for molecular research in *Miscanthus*. Genomic study of *Miscanthus* is currently focused on the construction of genetic maps using AFLP [7], SSR [8], RNA-seq [9], and GBS (genotype by sequencing) [10].

Expressed sequence tags (ESTs) are frequently useful for plant species that do not have sufficient genomics resources, providing functional profiles of genes as well as a basis of evolutionary study. Owing to massively parallel sequencing technologies, large numbers of ESTs can be easily generated under different growing conditions or tissue types; however, only five *Miscanthus* ESTs are publicly available in the NCBI’s dbEST as of December, 2013 (http://www.ncbi.nlm.nih.gov/dbEST).

In the current study, we generated transcriptome data sets from *M. sacchariflorus* and *M. sinensis*, the suspected progenitors of the *M. × giganteus* species which is preferred for biomass production. In particular, we chose those two species to scan transcriptomes because they will be served as mapping parents and the collection of gene profile may be necessary in the future work. We determined functional profiles of transcriptome sets from both leaf and rhizome for each *Miscanthus* species and compared the profiles. *M. sinensis* is known to have weak (or sometimes no) rhizomes but *M. sacchariflorus* has vigorous rhizomes, so it was of interest to identify genes showing significantly different expression in rhizomes than other tissues [11]. In addition, using the massive transcriptome data, we estimated the time of divergence among the two *Miscanthus* species, sorghum and *Saccharum*.

## Results and discussion

### 454 sequencing and de novo assembly of *Miscanthus* EST

**Figure 1:**
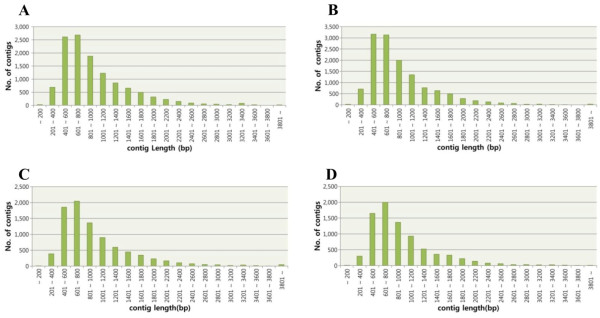
**Size distribution of the contigs assembled from *M. sinensis *** **leaf (A) and rhizome (B), and *M. sacchariflorus *** **leaf (C) and rhizome (D) tissues.**

In *M. sinensis*, a total of 457,891 and 512,950 reads from leaf and rhizome cDNA libraries were generated, spanning about 140 mega base pairs (Mbps) and 155 Mbps, respectively. The numbers of reads from the leaf (288,806) and rhizome (267,952) libraries in *M. sacchariflorus* were slightly less than those in *M. sinensis*; however, the average read length was longer than in *M. sinensis* (Table 1). After the assembly of raw reads (Materials and Methods for details), the leaf (MSIL, hereafter) and rhizome (MSIR) cDNA libraries of *M. sinensis* generated a total of 12,166 and 13,170 contigs, respectively. The leaf (MSAL) and rhizome (MSAR) libraries in *M. sacchariflorus* have 8,731 and 8,104 contigs, respectively. It is no surprise that the number of reads is positively correlated with that of contigs. The average numbers of reads in a contig are 28.4, 28.6, 23.6, and 23.4 for MSIL, MSIR, MSAL, and MSAR, respectively. The length distribution of the contigs is shown in Figure 1. The assembly produced a substantial number of large contigs in *M. sinensis* (12,144 for leaf and 13,146 for rhizome were ≥ 200 bp in length). Likewise, 8,721 and 8,095 contigs were assembled to more than 200 bp in MSAL and MSAR, respectively. The average contig lengths were 923 (MSI) and 993 bp (MSA) while the average singleton lengths were 281 (MSI) and 303 bp (MSA). The genome-wide coverage of unigenes based on the size of the haploid genome of *M. sinensis* and *M. sacchariflorus*[12] was about 1.5% and 1.1%, respectively, while the estimated coverage of coding sequences in sorghum was 5.5% [6]. However, genome size is not consistent with the number of genes (C-value paradox). The haploid genome size of *Miscanthus* spp. is about three times bigger than that of sorghum, in part due to the whole genome duplication event after its divergence from the sorghum lineage [8-10]; thus, simple comparison of coding sequences to each genome may not be able to explain the coverage of our ESTs to total transcriptomes in *Miscanthus*. For example, a total of 34,355 (MSI) and 35,177 (MSA) unigenes (out of contigs and singletons combined in Table 1) have significant similarities to sorghum gene models (34,496 in total) [6] based on blast search (*e* < 10^-5^), indicating that the number of *Miscanthus* ESTs presented in this study is sufficient for further analysis.

**Table 1:** **Sequencing and assembly statistics for *Miscanthus sinensis *** **and *M. sacchariflorus *** **ESTs**

	***M. sinensis***		***M. sacchariflorus***	
	**Leaf**	**Rhizome**	**Leaf**	**Rhizome**
Read No.	457,891	512,950	288,806	267,952
Total read length (bp)	140,496,482	155,223,677	96,218,568	89,406,409
Average read length (bp)	307	303	333	333
Contig No	12,166	13,170	8,732	8,104
Contig length (bp)	11,802,712	12,154,482	8,670,971	7,832,874
Average length of contig (bp)	970	923	993	967
Contig range (bp)	84-5,164	100-6,590	125-6,402	123-6,230
> = 1 kb	4,280 (35.2%)	4,149 (31.5%)	3,079 (35.3%)	2,782 (34.3%)
> = 200 bp	12,144 (99.8%)	13,146 (99.8%)	8,721 (99.9%)	8,095 (99.9%)
Singleton No.	89,648	112,138	66,881	63,212
Singleton length (bp)	25,195,941	31,614,461	20,040,968	19,174,280

### Functional annotation of ESTs

**Figure 2:**
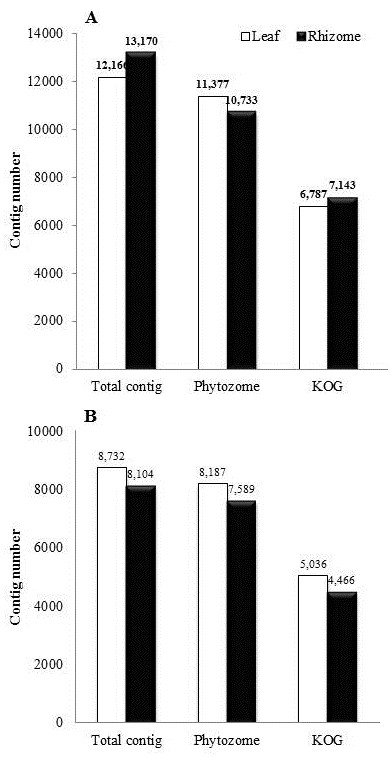
**Numbers of contigs similar to transcripts deposited in Phytozome and KOG databases.** The similarity is determined based on the BLAST search (*e* < 10^-5^) of contigs from *M. sinensis***(A)** and *M. sacchariflorus***(B)**.

In order to assign putative functions to ESTs, only contigs were subjected to similarity searches because short singletons could yield false positive results. First, contigs were blasted against all the genome sequences in major biological databases such as Phytozome [13] and KOG (euKaryotic Orthologous Group) [14] with a cutoff e-value of 10^-5^ (Figure 2). A total of 93% (11,377) of the total MSIL contigs and 81% (10,733) of the total MSIR contigs matched Phytozome transcripts from one or more of twenty-five plant species, while 94% of MSAL and MSAR contigs had significant hits. Contigs from rhizomes had relatively fewer matches (the number of hits in a specific database/total number of contigs obtained from *Miscanthus*) than those from leaves, consistent with limited sequence information available from rhizomatous plants and limited knowledge of rhizome-specific gene functions. Next, we blasted the contigs against the KOG in order to determine conserved orthologous genes. MSIL and MSIR contigs had 56% and 54% significant hits against the KOG versus 58% and 55% for MSAL and MSAR, respectively. *M. sacchariflorus* shows higher proportions than *M. sinensis* because of the smaller number of contigs. In addition, since the KOG for plant genes was constructed solely based on *Arabidopsis thaliana*, the ~40% of contigs which do not have significant matches to genes in the DB may reflect monocot-dicot divergence or lineage- or species-specific genes.

**Figure 3:**
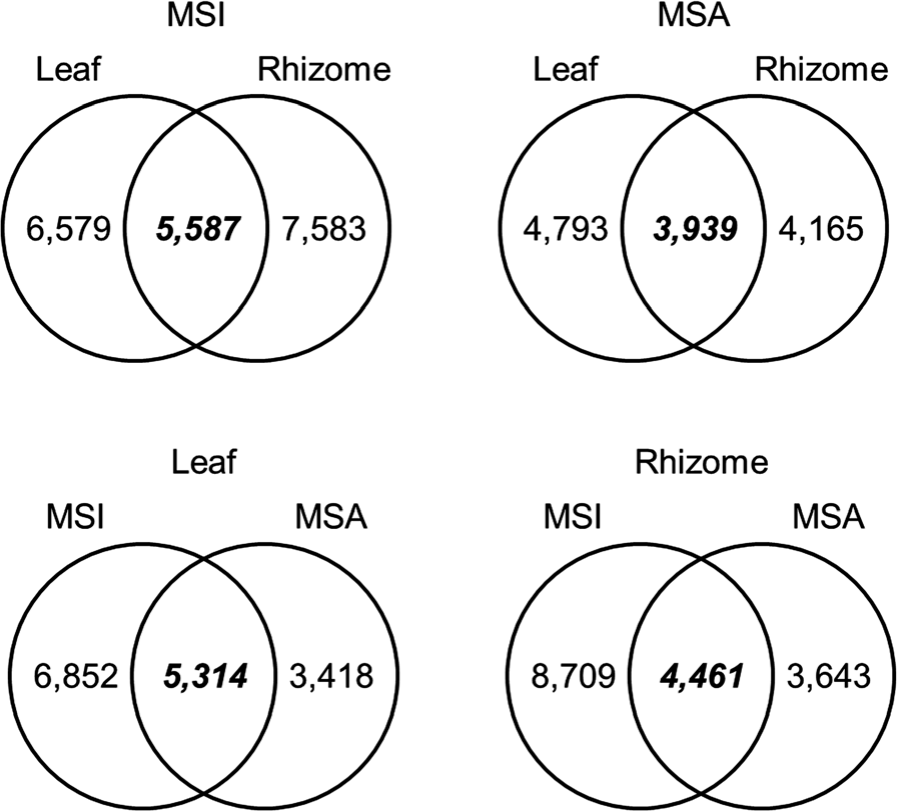
**The number of highly similar sequences between consensus groups.** The numbers in *italic* represents highly similar sequences between two groups. For example, there are 5,587 highly similar consensus sequences between two groups, leaf and rhizome, in *M. sinensis*. MSI: *M. sinensis*; MSA: *M. sacchariflorus*.

More than 50% of transcripts were specific to either leaf or rhizome libraries. Highly similar leaf and rhizome contigs were determined by BLASTN (e < 10^-10^) to calculate proportions of genes expressed in both organs (Figure 3). In *M. sinensis*, 5,587 genes were expressed in both leaf and rhizome, comprising 45.9% and 42.4% of MSIL and MSIR, respectively. In *M. sacchariflorus*, 3,939 contigs were expressed in both tissues comprising 45.1% and 48.6% of MSAL (8,732) and MSAR (8,104), respectively.

More than 39.1% of transcripts were specific to *M. sinensis* or *M. sacchariflorus* libraries. The leaf libraries of the two *Miscanthus* species had 5,314 highly similar contigs which was 43.7% in MSIL and 60.9% in MSAL whereas the rhizome libraries had the 4,461 contigs whose proportion in MSIR and MSAR were 33.9% and 55.0%, respectively. The relatively higher proportions of highly similar contigs between *M. sacchariflorus* and *sinensis* in *M. sacchariflorus* than in *M. sinensis* libraries might be caused by less number of contigs in *M. sacchariflorus* and that is consistent with the higher proportion of well conserved orthologous genes in *M. sacchariflorus* library than *M. sinensis* (Figure 2).

A total of 2,744 transcripts were found to be expressed in all tissue types and species. As expected, GO classification suggested that those transcripts were mostly related to basal plant metabolic processes or structures. Although a large number of transcripts seem to be tissue- or species-specific genes as shown in this work, housekeeping functions appear well conserved in different tissues and species.

### Comparative analysis of rhizome-enriched genes of *Sorghum* spp. to *Miscanthus* ESTs

**Figure 4:**
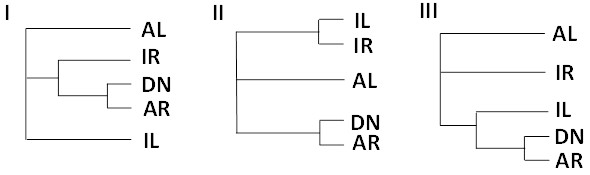
**Phylogenetic trees in which rhizome-enriched genes of *S. halepense *or *S. propinquum *(DN) and ESTs of *M. sacchariflorus *(AR) are grouped together.** The genes included in these trees are further analyzed as rhizome-enriched genes in *Miscanthus*. DN: rhizome-enriched genes of *S. halepense* or *S. propinquum*; IL: leaf ESTs of *M. sinensis*; IR: rhizome ESTs of *M. sinensis*; AL: leaf ESTs of *M. sacchariflorus*; AR: rhizome ESTs of *M. sacchariflorus*.

Previously, rhizome-enriched genes have been identified from *S. halepense* and *S. propinquum*[15]. Similarity search showed that 383 out of 768 rhizome-enriched genes found in the two *Sorghum* species have at least one ortholog in *Miscanthus* ESTs and 171 have orthologs in both leaf and rhizome from the two *Miscanthus* species. The 171 rhizome-enriched genes with four corresponding orthologs in *Miscanthus* (*Sorghum* orthologs in the four libraries) were defined as orthologous groups and a phylogenetic tree was generated for each orthologous group. Since *M. sinensis* is known to have weak or no rhizomes whereas *M. sacchariflorus* has vigorous rhizomes, only three types of trees clustering rhizome-enriched genes of the two sorghum species and rhizome ESTs of *M. sacchariflorus* (DN and AR in Figure 4, respectively) were further analyzed although 15 different topologies were generated. Interestingly, no tree topology showed clustering of rhizome-enriched genes from the two sorghum species and rhizome ESTs of *M. sinensis*, suggesting that rhizome-enriched genes from the two *Sorghum* species are more similar to the rhizome ESTs of *M. sacchariflorus*. The three topologies comprised 31 orthologous groups. Table 2 shows ESTs included in the 31 orthologous groups with their putative functions. The genes included in those three topologies were annotated by gene ontology (GO). While no particular category was predominant, the ‘response to stimulus’ (GO:0050896), seemingly related to signals in response to stress, occupies a high portion. Jang *et al.*[15] suggested that the loss of rhizomatousness in *S. bicolor* might have been caused by changes in gene regulation. Although the 31 genes are not specifically associated with functions and GO categories found in roots or rhizomes, these genes could be primary targets for investigating rhizomatousness in *Miscanthus*. The 31 genes are all expressed in both leaf and rhizome in the two *Miscanthus* species, but rhizome ESTs of *M. sacchariflorus* are still closer to rhizome-enriched genes from the two sorghum species, which could be caused by regulatory changes in the upstream regions of those genes. However, there are a number of ESTs exclusively expressed in *Miscanthus* rhizomes (Figure 3). It remains to be determined whether they are truly specific to rhizomes or due to inadequate sampling in random sequencing of transcriptomes.

**Table 2:** **Putative functions of genes included in the three orthologous groups shown in Figure**4

		***M. sacchariflorus***		***M. sinensis***		
**Groups**	**DN #**	**Leaf (AL)**	**Rhizome (AR)**	**Leaf (IL)**	**Rhizome (IR)**	**Putative functions**
I	DN551695	isotig00853	isotig08093	isotig00083	isotig03753	Polyubiquitin
	DN552466	isotig05764	isotig07409	isotig05877	isotig08182	Serine-threonine kinase receptor-associated protein
	DN552491	isotig03800	isotig03933	isotig09327	isotig06471	Heat shock protein 90
	DN552676	isotig01244	isotig07514	isotig10039	isotig01837	kh domain containing protein
	DN552708	isotig05041	isotig04880	isotig07298	isotig07578	Catalase
	DN552740	isotig04698	isotig00757	isotig03722	isotig02800	rna-binding protein
II	DN551779	isotig00796	isotig01314	isotig01678	isotig04296	Amino acid expressed
	DN551936	isotig05246	isotig06286	isotig03192	isotig09807	Actin
	DN552433	isotig06434	isotig05172	isotig07270	isotig07933	at1g67350-like protein
	DN552562	isotig01135	isotig05901	isotig08694	isotig08499	Poly -binding protein
	DN552570	isotig06844	isotig02287	isotig05455	isotig11299	at5g02460-like protein
	DN552671	isotig06879	isotig05162	isotig09810	isotig07725	Plasminogen activator inhibitor 1 rna-binding protein
	DN552679	isotig02638	isotig06563	isotig01634	isotig00900	Acyl carrier protein 3
	DN552709	isotig03948	isotig03636	isotig02927	isotig02248	Ras-related protein rab11c
	DN552742	isotig06246	isotig03810	isotig02007	isotig05233	No hits found
	DN552766	isotig02679	isotig05804	isotig03934	isotig03115	Membrane protein
	DN552772	isotig02777	isotig06530	isotig04172	isotig09766	S-adenosylmethionine decarboxylase
III	DN551750	isotig02250	isotig03324	isotig02111	isotig04464	Dolichyl-diphosphooligosaccharide-protein glycosyltransferase
	DN551751	isotig01665	isotig00318	isotig00973	isotig00230	40s ribosomal protein s14
	DN551768	isotig03736	isotig03218	isotig04157	isotig01466	No hits found
	DN551788	isotig05403	isotig05029	isotig05339	isotig04880	Reversibly glycosylated polypeptide
	DN551793	isotig00295	isotig00036	isotig00847	isotig00643	60s acidic ribosomal protein p1
	DN551798	isotig00508	isotig00357	isotig02756	isotig02156	nc domain-containing protein
	DN551841	isotig07246	isotig06497	isotig04474	isotig10222	Maize proteinase inhibitor
	DN551904	isotig03373	isotig04561	isotig01269	isotig04168	Ubiquitin-like protein smt3
	DN552392	isotig00059	isotig01841	isotig03321	isotig00727	No hits found
	DN552427	isotig07628	isotig07842	isotig01782	isotig09528	atp synthase gamma
	DN552437	isotig01515	isotig04375	isotig03437	isotig02624	Actin-depolymerizing factor 6
	DN552472	isotig01693	isotig01301	isotig00608	isotig00373	nac2 protein
	DN552626	isotig01047	isotig00215	isotig00478	isotig04878	60s ribosomal protein l26-1
	DN552650	isotig07218	isotig06635	isotig04233	isotig03259	40s ribosomal protein s15a

Groups indicate the three topologies of phylogenetic trees described in the figure. DN numbers indicate the NCBI accession numbers of rhizome-enriched genes from *S. halepense* or *S. propinquum*. Putative functions were predicted based on the best blastx hits of rhizome-enriched genes from *S. halepense* or *S. propinquum* against the NCBI’s non-redundant protein database.

### Speciation of *Miscanthus* spp., sugarcane, and sorghum

**Figure 5:**
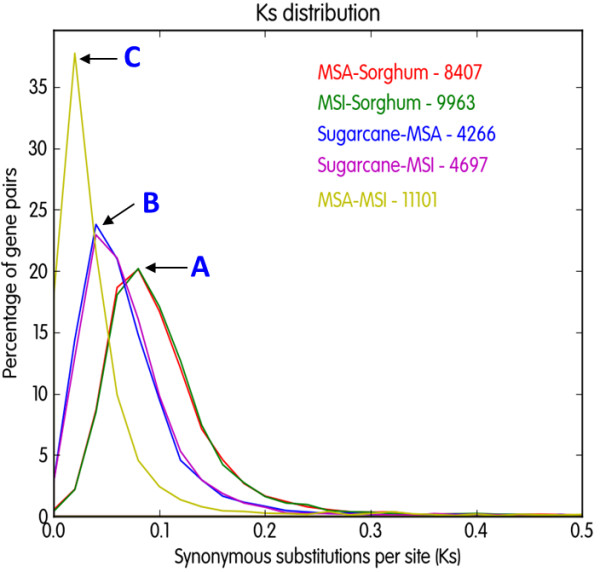
**Distributions of synonymous nucleotide substitution (Ks) values among tested grasses.** The numbers next to species names indicate the number of orthologous sequences used for plotting Ks distributions. Each peak represents time points of speciation between two species. **(A)** Speciation of two subtribes, Sorghinae and Saccarinae based on the comparison of MSA – sorghum and MSI – sorghum (Ks = 0.08). **(B)** Speciation of *Saccharum* and *Miscanthus* genera based on MSA – sugarcane and MSI – sugarcane (Ks = 0.04). **(C)** Speciation between two *Miscanthus* species based on MSA-MSI (Ks = 0.02). MSA; *M. sacchariflorus*, MSI; *M. sinensis*.

Synonymous nucleotide substitution (Ks) of orthologous gene pairs provided insight into the timing(s) of divergence among these grasses (Figure 5). Considering 6.5 × 10^-9^ synonymous changes per synonymous site per year as the neutral mutation rate in monocots [16], the divergence of Sorghinae and Saccharinae subtribes (peak A) was 0.08, corresponding to 6.2 million years ago (MYA). Divergence between *Saccharum* and *Miscanthus* genera (peak B, Ks = 0.04) was estimated as 4.6 MYA; and between the two *Miscanthus* species (peak C, Ks = 0.02) as 1.5 MYA.

The divergence between sugarcane and sorghum has previously been investigated using orthology, with multiple studies estimating that the two species diverged about 7.7 - 9.0 MYA [3,17,18]. Since *Miscanthus* and sugarcane belong to the same subtribe (Saccharinae) and share a common ancestor, the estimation may be consistent with the case which sorghum and *Miscanthus* are compared; however, our estimation in was about 1.4 - 2.8 million years more recent (6.2 MYA). The discrepancy from previous reports may be due to different sequence data used for the comparison. Kim *et al.*[18] and Wang *et al.*[3] estimated the divergence time at 7.7 MYA but Jannoo *et al.*[17] reported the divergence time at 8.0 - 9.0 MYA. The former two studies used sugarcane ESTs mostly originating from hybrid sugarcane cultivar(s) but the latter used only a single gene, *Adh1,* to estimate the divergence between the two subtribes. The sugarcane genome is more complex than *Miscanthus* owing to a higher ploidy number and hybrid sugarcane is still more complex because of its interspecific origin and frequent aneuploidy caused by its breeding history [19]. Therefore, although the common ancestor of *Miscanthus* and sugarcane diverged from the sorghum lineage at the same time, hybrid sugarcane could be highly polymorphic within single cultivars, and appear more diverged from sorghum than *Miscanthus*, resulting in the overestimation of Ks values. We also infer that sugarcane and *Miscanthus* share a common ancestor about 4.6 MYA. Due to lack of nucleotide information in *Miscanthus*, its divergence from the *Saccharum* lineage had not been estimated previously [18]. In turn, the estimation of the two genera could be overestimated because of the ESTs from hybrid sugarcane cultivar(s). However, our estimation together with EST sequences may serve as a guideline for future research in the evolution of *Miscanthus* species until a detail of sugarcane genome is released.

Recently published genetic maps revealed that the *Miscanthus* lineage experienced whole genome duplication after its divergence from sorghum [8,10]. Although we tried to find paralogs from the EST datasets to date the duplication, signals were mostly hidden or too weak. Finding recent whole genome duplication events using ESTs is somewhat difficult in that paralogous and redundant sequences are not clearly identifiable in EST datasets and recent whole genome duplication signals are frequently masked by recent single gene duplication signals. If the whole genome duplication occurred in *Miscanthus* after its divergence from a common ancestor shared with sorghum (ca. 6.2 MYA), two hypotheses are highly likely: (1) the *Miscanthus* genome had experienced some ‘diploidization’ (loss of one member of duplicated gene pairs) before the two *Miscanthus* species diverged (ca. 1.5 MYA), and (2) the (diploid) *M. sinensis* genome experienced appreciable diploidization after the two *Miscanthus* species diverged. If the former is true, *M. sacchariflorus* might have had an additional genome-wide duplication after the two species diverged. Considering the recent divergence of the two species (1.5 MYA), the latter may be more plausible. However, this has to be elucidated with additional information.

### Functional differentiation of *Miscanthus* and sorghum genes

**Figure 6:**
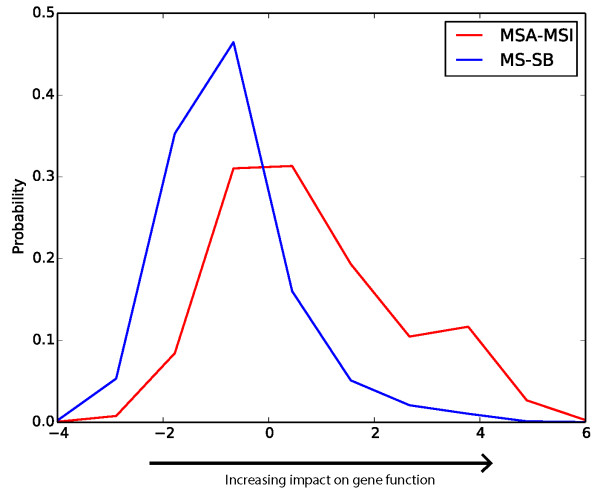
**Distribution of nsSNP impact on protein function in the sorghum and *Miscanthus *lineages.** Red line represents nsSNP between the two *Miscanthus* species. Blue line depicts nsSNPs between *Miscanthus* and sorghum.

Conserved protein domains are frequently essential to the function of a gene. Amino acid change that occurs in the conserved regions of a protein sequence is more likely to have larger impact on gene function than changes in other regions [20]. The impacts of nsSNPs on gene functions were predicted based on gene conservation profiles as described by Paterson *et al.*[21]. A total of 43,770 nsSNPs were identified between *Miscanthus* and sorghum and 35,818 nsSNPs between MSA and MSI. If a base involved in a specific nsSNP site is predominant in the 30 published genomes (see Materials and Methods), the base is defined as a common allele. Otherwise, it was defined as a rare allele. In Figure 6, the X-axis indicates the impact on gene function. If an nsSNP is changed from a common to a rare allele, it has positive value. Therefore, the impact of nsSNPs on gene function increases from negative to positive values. The Y-axis represents the probability of nsSNPs with different impact. The impacts on gene function of SNPs that differ between the two *Miscanthus* species are significantly more positive than SNPs between sorghum and *Miscanthus* (t statistics = 125.37, *P* = 0), indicating that striking mutations are more frequently found between two *Miscanthus* species than sorghum and *Miscanthus*. This may be a consequence of the much higher level of gene duplication in *Miscanthus*, in which striking mutations may be better tolerated by virtue of the presence of a second gene copy that may confer essential functionality.

Table 3 shows the ten largest-effect nsSNPs that are found between reference (sorghum) and mutated alleles (*Miscanthus*). The ten genes could be classified into five functional categories which are ABC transporters, glycogen synthase kinase-3, ATPase, beta tubulin, and cellulose synthase. Interestingly, except for cellulose synthase (Sb01g019720), three functions (ABC transporter, ATPase, and glycogen synthase kinase-3) and beta tubulin have very close GO terms, ATP binding (GO:0005524) and GTP binding (GO:0005525), respectively. These genes may be of interest for further functional investigation.

**Table 3:** Top 10 nsSNPs having large impacts on gene functions

**Gene**	**Position**	**Ref AA**	**Mut AA**	**Function**
Sb10g004070	691	W	H	Pleiotropic drug resistance proteins, ABC superfamily
Sb01g043620	323	V	I	Calcium transporting ATPase
Sb01g019720	694	E	Q	C1-like domain|Cellulose synthase
Sb01g006310	193	V	I	Beta tubulin
Sb04g008580	231	G	S	Glycogen synthase kinase-3
Sb10g004070	688	Q	S	Pleiotropic drug resistance proteins, ABC superfamily
Sb03g027480	801	M	I	Pleiotropic drug resistance proteins, ABC superfamily
Sb03g027490	849	M	I	Pleiotropic drug resistance proteins, ABC superfamily
Sb04g008580	252	A	E	Glycogen synthase kinase-3
Sb10g004070	692	I	G	Pleiotropic drug resistance proteins, ABC superfamily

Reference (Ref AA) and mutation amino acid (Mut AA) indicate sorghum allele and mutated allele in *Miscanthus*, respectively.

## Conclusions

The current study provides nearly 227,000 ESTs and 146,000 ESTs from *M. sinensis* and *M. sacchariflorus*, respectively, greatly enriching knowledge of the transcriptome of this promising lignocellulosic bioethanol crop. *M. sinensis* and *M. sacchariflorus* are of particular interest because they are suspected progenitors of the triploid *M. × giganteus* which is cultivated as a biomass crop. These ESTs can be utilized in many ways such as seeking molecular markers, gene prediction in genome sequences, and gene expression studies. A core set of (largely housekeeping) genes are expressed in common in the two *Miscanthus* species and the two tissues studied, with large populations of genes that appear to be tissue specific. Genes showing rhizome-enriched expression in rhizomatous *Sorghum* species appear to correspond more closely to those in *M. sacchariflorus* which has aggressive rhizomes, than those in *M. sinensis* that has weak rhizomes. The analysis of nsSNPs showed that striking mutations are more frequently found between two *Miscanthus* species than between *Miscanthus* and sorghum, reflect that *Miscanthus* seems to be more tolerant of point mutations than sorghum. A plausible explanation of this observation is that genomic redundancy resulting from its recent genome doubling has relaxed selection on duplicated gene copies. We also analyzed ESTs to make preliminary inferences about *Miscanthus* evolution. As stated in the text, *Miscanthus* experienced whole genome duplication since its divergence from sorghum. Although ESTs generally provided too little information to clearly identify paralogs which were formed by the whole genome duplication, they were still useful to predict speciation events by providing clear signal of orthologous relationships. The exact timing of the whole-genome duplication event can be evaluated when additional genome sequence and detailed maps are available, and our ESTs will be able to contribute to such detailed evolutionary studies.

## Methods

### Plant materials and growth conditions

*M. sinensis and M. sacchariflorus* were collected in South Korea, and their ploidy levels and morphological identities were determined as in a previous report [22]. Plants were grown for a year in plastic pots and placed in the greenhouse at Mokpo National University, Mokpo, South Korea. No fertilizer was applied until sampling, but watering was given when the pot soil dried. The greenhouse temperature was controlled from 24 to 28°C during the growth period without supplemental light. Leaves and rhizomes were sampled in the vegetative state before flowering. Newly developed rhizomes (<1 year old) along inner surface of the pot were harvested and pooled after grinding. All samples were sent to the National Instrumentation Center for Environmental Management (NICEM, Seoul, South Korea) for cDNA library construction.

### Construction of cDNA library and sequencing of the library

Total RNA was isolated from rhizome and leaf tissues of *M. sinensis* and *M. sacchariflorus* using Rneasy Plant Mini Kit (Qiagen, Seoul, Korea) according to the manufacturer’s instructions. Extracted RNA samples were quantified and quality-checked using a Bioanalyzer 2100 (Agilent Technologies, Santa Clara, CA). Total RNA (6 μg) was reverse transcribed using Super Script II (Life Technologies, Carlsbad, CA). Second strand cDNA was synthesized using Advantage 2 Polymerase Mix (Clontech, Seoul, Korea). cDNA samples were purified using QIAquick PCR purification kit (Qiagen, Seoul, Korea). The purified fragments were then used to create the 454 single-stranded cDNA library using a 454 library preparation kit (Roche). The fragment ends were polished using T4 ligase and T4 polynucleotide kinase and adaptors containing primer sequences and a biotin tag were ligated to the fragment ends (Roche, Brandford, CT). The fragments with properly ligated adaptors were immobilized onto magnetic streptavidin-coated beads (Roche, Brandford, CT). Nicks or gaps between the adaptors and the dscDNA fragments were repaired using the fill-in polymerase. The non-biotinylated strands of the immobilized dscDNA fragments were melted off to generate the single-stranded cDNA library for 454 sequencing.

### Assembly of 454 reads and annotation of contigs

Before assembly, extended multiplex identifier (MID) sequences of 3′ and 5′ ends, poly A(T) tail, short sequences (<50 bp) and low complexity were removed. Four EST data sets from leaf and rhizome tissues of two *Miscanthus* species were individually assembled using GS *de novo* assembler version 2.6 (Roche Diagnostics Corporation, http://454.com/products/analysis-software/index.asp) with default parameters. From the assembly results, only consensus sequences (contigs) were taken for further analyses due to the short length of singletons. Functional annotation was performed by sequence comparison with public databases. All contigs were blasted against the Phytozome database (http://www.phytozome.org, version 7.0, April 2011) which includes annotated genes with the KOG assignments (*e* < 10^-5^).

### Comparison to rhizome-enriched genes from *S. halepense* and *S. propinquum*

A total of 768 ESTs previously reported as derived from rhizome-enriched genes in *S. halepense* or *S. propinquum*[11] were blasted against *Miscanthus* ESTs to find putative orthologs (*e* < 10^-20^). The putative orthologs from five different datasets - rhizome and leaf ESTs from two *Miscanthus* species and rhizome-enriched genes from *Sorghum* spp. - were used to construct phylogenetic trees. The five orthologs were multiple-aligned by ClustalW [23]. ClustalW was also implemented for generating phylogenetic trees using the Neighbor Joining algorithm. The phylogenetic trees were grouped based on their topologies by determining symmetric differences among trees with the Treedist function in the PHYLIP package [24], in order to compare rhizome-enriched genes from the two *Sorghum* species to ESTs from *Miscanthus*. The phylogenetic trees were grouped based on their topologies, in order to infer rhizome-enriched ESTs in *Miscanthus* species. For the candidate rhizome-enriched ESTs in *Miscanthus*, putative functions were classified using Blast2GO [25].

### Estimation of divergence time in Saccharinae subtribe

To estimate the speciation time among *M. sacchariflorus*, *M. sinensis*, sugarcane, and sorghum, Ks values between orthologous ESTs were calculated. Sugarcane ESTs and sorghum gene sequences were obtained from dbEST (http://www.ncbi.nlm.nih.gov/dbEST/) and Phytozome (http://www.phytozome.net/), respectively. To identify putative orthologs among species, each sequence from one species was blasted against all sequences from other species and the best hits were taken. A pair of best hit was defined as putative orthologs when the pair was aligned over 300 bp or more (*e* < 10^-20^). Each member of a pair of sequences was subjected to blastx search against predicted sorghum proteins. The best match was considered significant if the alignment length was more than 100 amino acids (*e* < 10^-15^). If no significant match was found, the pair of sequences was excluded from further analysis. The cleaned pairs of sequences were translated using the Genewise program, which can take frameshift sites into consideration [26], with the corresponding best match protein in *S. bicolor* as reference. For each pair of paralogs, the two translated products were aligned using ClustalW [23], and the resulting alignment was used as a guide to align the nucleotide sequences. After removing gaps and N-containing codons, the level of synonymous substitution was estimated using the maximum likelihood approach implemented in the program CODEML, which is a part of the PAML package [27]. Batch jobs were performed using in-house python scripts.

### Nonsynonymous SNP analysis

Orthologous protein sequences from *S. bicolor*, *M. sacchariflorus* and *M. sinensis* previously defined were used to identify nsSNP. To find nsSNPs between MSA and MSI, pairwise alignment of predicted proteins were conducted. For nsSNPs between sorghum and *Miscanthus*, the exactly same proteins across the two *Miscanthus* species were aligned to sorghum proteins. The identified nsSNPs were classified into two groups: One group contains nsSNP that is polymorphic between MSA and MSI. nsSNPs in the other group are between sorghum and the two *Miscanthus* species. Protein sequences from the 30 published genomes (http://chibba.agtec.uga.edu/duplication/) were clustered and aligned using orthoMCL [28] and ClustalW [23]. The nsSNPs identified from the three species were aligned to the orthologous gene clusters. The impact of nsSNPs on gene function is evaluated by functional impact score (FIS) using the formula described by Paterson *et al.*[21].

### Availability

All the ESTs are downloadable at the NABIC (National Agricultural Biotechnology Information Center; http://nabic.rda.go.kr/) with accession numbers indicated; NN-0642-000001 (MSAL), NN-0643-000001 (MSAR), NN-0639-000001 (MSIL), and NN-0641-000001 (MSIR).

## Abbreviations

AFLP: Amplified fragment length polymorphism; SSR: Simple sequence repeat; EST: Expressed sequence tag; MYA: Million years ago; MID: Multiplex IDentifier; GO: Gene ontology; MSI: *M. sinensis*; MSA: *M. sacchariflorus*; MSIL: *M. sinensis* leaf library; MSIR: *M. sinensis* rhizome library; MSAL: *M. sacchariflorus* leaf library; MSAR: *M. sacchariflorus* rhizome library; nsSNP: Nonsynonymous single nucleotide polymorphism; Ks: Synonymous nucleotide substitution.

## Competing interests

The authors declare that they have no competing interests.

## Authors’ contributions

AHP, DSK, GJL conceived the study. CK, THL, AHP, GJL analyzed results and wrote the paper. YJJ, SJC performed experiments. DSK provided materials. THL, HG provided bioinformatics support and discussion. All authors read and approved the final manuscript.
